# The South African Rugby Injury and Illness Surveillance and Prevention Project (SARIISPP): SARU Boys’ Youth Week Injury Surveillance Report, 2023

**DOI:** 10.17159/2078-516X/2025/v37i1a21508

**Published:** 2025-04-15

## Executive Summary

As part of the South African Rugby Union (SARU) Injury and Illness Surveillance and Prevention Project (SARIISPP), SARU records and investigates injury data from the annual SARU Youth Week tournaments. The BokSmart National Rugby Safety Programme has gathered and analysed these data since 2011 for the SARU Boys’ Youth Week tournaments.

This report focuses on two 2023 Boys’ Rugby tournaments: the Grant Khomo Under 16 Week (GKu16) and Craven Week Under 18 (CWu18). The two tournaments consisted of 36 teams, and 54 matches were played. Comparisons are made between these two SARU Boys’ Youth Week tournaments in 2023 (GKu16 vs CWu18) and between 2011 and 2023. It must be noted that no GKu16 and CWu18 tournaments were held in 2020 and 2021 due to the COVID-19 restrictions. Additionally, no data were collected at the 2022 GKu16 and CWu18 tournaments due to the continued financial impact of the COVID-19 pandemic on the programme’s resources.

Each medical facility at the SARU Youth Week tournaments has a designated researcher(s) on-site, who, together with the tournament medical doctors, records and documents the tournament injury data daily. The analysis then investigates the injury patterns for the SARU Boys’ Youth Weeks (GKu16 vs. CWu18). Furthermore, the analysis compares the profiles of injured players at each tournament. During the investigation of these patterns, potential areas of concern, such as changes in the game, tournament structure or medical support services, can be identified. Consequently, injury-specific interventions can be developed and implemented where the evidence supports such a need.

In 2023, 59 time-loss injuries were recorded for both tournaments combined (GKu16 and CWu18). This equated to an average injury rate of 34 (25 to 43) injuries per 1000 player hours; data are expressed as mean (±95% confidence intervals) injuries per 1000 player hours. The Time-Loss injury incidence for the GKu16 and CWu18 tournaments was 40 (27 to 53) injuries per 1000 player hours and 27 (16 to 39) injuries per 1000 player hours, respectively. Combining the injury incidence data collected over the ten included years, there was no significant difference between these two age groups. However, the CWu18 tournament tended to have a lower injury incidence.

In 2023, *Tackling* (tackler) was the most frequent injury-causing event, followed by *Being Tackled* (ball carrier), and then *Running-related injury mechanisms*. *Tackling front-on (regulation)* and *Tackling side-on (regulation)* were the most frequent injury-causing mechanisms involved in the Tackler injuries. While *Being Tackled front-on (regulation)* and *Being Tackled from behind (regulation)* / *Being Tackled side-on (regulation)* were the most frequent injury-causing mechanisms involved in Ball Carrier injuries. Lastly, *Cutting manoeuvres/changing direction* was the most frequent injury-causing mechanism in *Running-related injuries*.

The most common injury type for the combined tournaments was *Central Nervous System* injuries. No difference was found between tournaments. *Head and Neck* was the most common injury location in 2023, accounting for 48% of the injuries, with 61% occurring in the GKu16. As expected, the incidence of *‘New’* injuries was higher than subsequent ‘*Recurrent’* injuries. Most muscle injuries were ‘*New*’ injuries, while joint/ligament injuries had comparatively more *‘Recurrent’* injuries than muscle injuries. Players who started the match sustained more injuries than those who joined the match as substitutions. *Hookers* and *Loose forwards* were the player positions with the highest normalised injury incidence per player per position for the combined tournaments.

There were 26 concussions recorded in the 2023 tournaments, a substantial increase from 2019. Furthermore, the act of *Tackling* (the tackler) contributed to 50% of the events causing concussions. Concussion rates have increased considerably since COVID-19; however, this increase was not significantly different.

Recommendations from this report advocate that the Boys’ rugby teams must focus on improving their tackling and ball-carrying techniques and increasing the coaching emphasis on developing, teaching, training, and preparing the players properly for executing safer contact techniques in match situations. Furthermore, running-related strength and conditioning, including cutting drills and drills incorporating changes of direction, were recommended to reduce running-related injuries.

**Figure:**
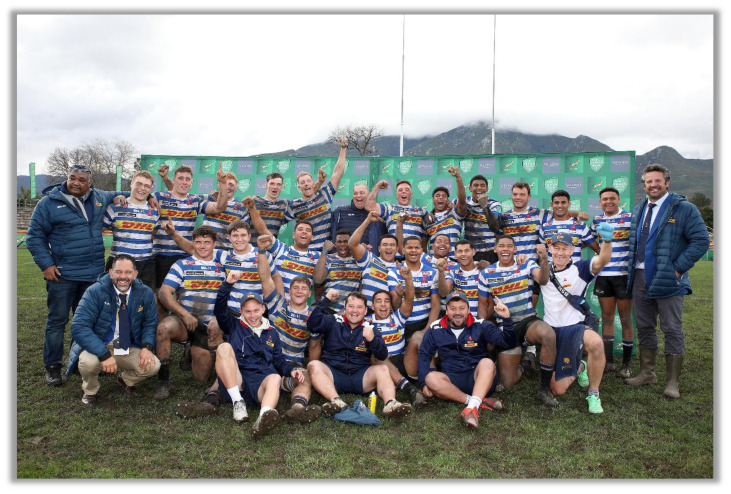


## Definitions

All definitions are based initially on the 2007 consensus statement for injury reporting in rugby union (1) and have since been realigned with the latest International Olympic Committee (IOC) consensus statement for methods of recording and reporting epidemiological data on injury and illness in sports (2).

### MEDICAL ATTENTION INJURY

All injuries seen by the tournament medical doctor or medical support staff were classified as Medical Attention injuries. These injuries are defined by the 2007 statement as an “*injury that results in a player receiving medical attention”* (1), and by the more recent IOC statement as *“a health problem that results in an athlete receiving medical attention”* (2).

### TIME-LOSS INJURY

Medical Attention injuries were further categorised as Time-Loss injuries, where appropriate, and defined by the 2007 statement as “*an injury that results in a player being unable to take a full part in future rugby training or match play*” (1). The IOC definition is *“a health problem that results in a player being unable to complete the current or future training session or competition”* (2). For clarity, this means an injury sustained by a rugby union player during a match or training session that prevented or would have prevented the player from taking full part in all rugby training activities and/or match play for more than one day following the day of injury, irrespective of whether match or training sessions were scheduled (3).

### INJURY RATE

This report defines an injury rate as the number of injuries expressed per 1000 player exposure hours. This method of expressing injury rate has been used in previous years’ Youth Week reports and other international literature, making comparisons easy. Moreover, the injury rate is expressed as a mean with 95% confidence intervals. A 95% confidence interval around a mean value indicates a 95% chance (i.e., very high chance) that the true value falls within this range. In this report, we present the 95% confidence intervals assuming a normal distribution of the data and use the approach of examining the overlap of the confidence intervals to determine whether the injury incidences are significantly different; if the range of confidence interval values of two comparisons does not overlap, there is a strong chance (95%) that their injury rates are different from each other. We have opted for this method because it is easy to use, conservative, and less likely to produce false positive results (4).

### NEW, SUBSEQUENT AND RECURRENT INJURIES

A ‘*New Injury’* was defined as when a player sustained his first injury. Any injury the *same* player sustained after this initial injury was defined as a *‘Subsequent Injury’.*

According to the IOC statement, any subsequent injury to the same site and of the same type is referred to as a ‘*Recurrence’* if the index injury was fully recovered before reinjury and as an *‘Exacerbation’* if the index injury was not yet fully recovered (2).

To provide more detail on subsequent injuries for practitioners, one can further categorise the subsequent injuries into one of four groups:

- Different site - Different type
- Different site - Same type
- Same site - Different type
- Same site - Same type

According to the 2007 Consensus Statement for rugby, any subsequent injury classified as ‘Same site - Same type’ was a *‘Recurrent injury’* (1).

**Figure:**
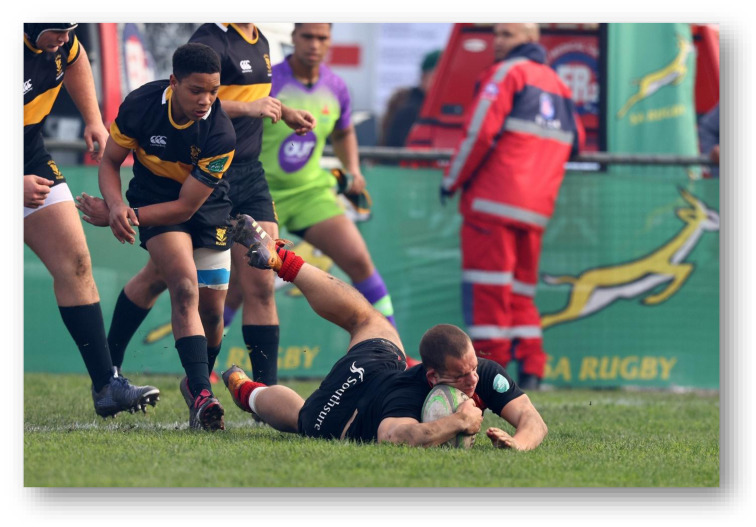


## Key Findings

### Injury Incidence

**Figure 1:**
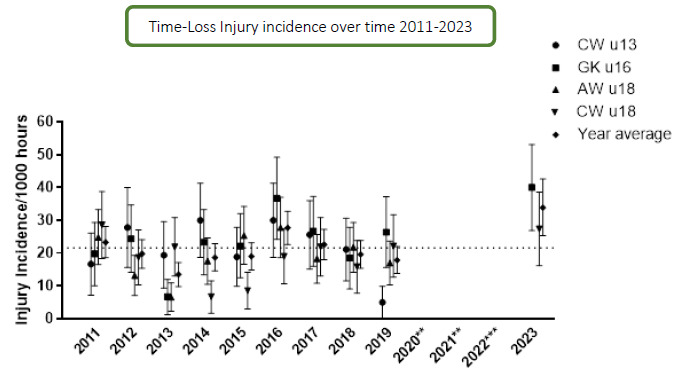
Injury incidence/1000 player hours and 95% confidence intervals of Time-Loss injuries for the SARU Boys’ Youth Week Tournaments from 2011 – 2023. The dotted line reflects the average incidence for all tournaments over all the included years. **No GKu16 and CWu18 tournaments were held in 2020 and 2021 due to COVID-19 restrictions. *** No data collection was completed in 2022 due to financial constraints.

Thirty-six teams competed in the 2023 SARU Boys’ Youth Week tournaments (GKu16 = 20 teams, CWu18 = 16 teams). A total of 54 matches were played in 2023, and 88 Medical Attention injuries were recorded during the tournaments; sixty-seven percent of these (n = 59) were Time-Loss injuries. The combined tournaments’ injury incidence and 95% confidence intervals for all Medical Attention injuries was 51 (40 to 61) injuries/1000 player hours, and for Time-Loss injuries was 34 (25 to 43) injuries/1000 player hours. The incidence of Medical Attention injuries and Time-Loss injuries in the GKu16 and CWu18 were not significantly different from each other in 2023 (Table 1). The average combined injury incidence for Time-Loss injuries was higher in the 2023 SARU Boys’ Youth Week tournaments than in the other years, although this difference was also not significant. The numbers of Medical Attention and Time-Loss injuries per match and per hour of match play across the tournaments are represented in Table 2. Figure 1 shows the pattern of Injury incidence/1000 player hours and 95% confidence intervals of Time-Loss injuries for each tournament across the years (2011 to 2023) (Figure 1).

**Figure 2:**
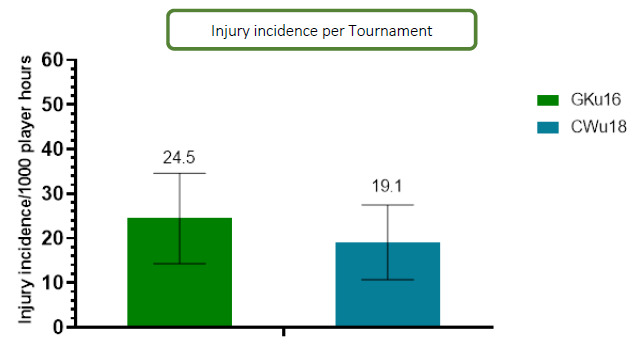
Injury incidence/1000 player hours and 95% confidence intervals at the GKu16 and CWu18 SARU Boys’ Youth Week tournaments from 2011 – 2023.

Further analysis was completed on Time-Loss injuries and for GKu16 and CWu18 tournaments only. The combined data from 2011 to 2023 shows a slight, albeit not significant, decrease in injury incidence going from GKu16 to CWu18. (Figure 2).

### Injury Incidence Trends

#### U16 Grant Khomo Week (GKu16)

**Figure 3:**
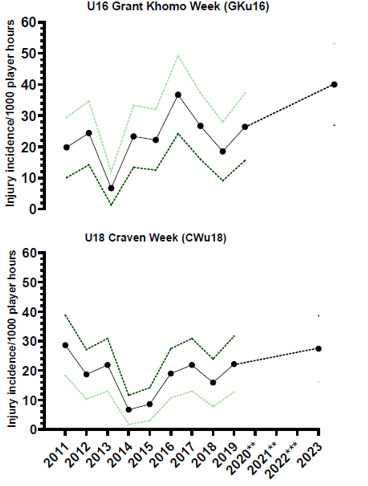
Time-Loss Injury incidence for each SARU Boys’ Youth Week tournament, per year, from 2011 – 2023, including the upper and lower 95% Confidence Intervals (95%CI). **No GKu16 and CWu18 tournaments were held in 2020 and 2021 due to COVID-19 restrictions. *** No data collection was completed in 2022 due to financial constraints.

Due to COVID-19, the GKu16 tournament was not held in 2020 and 2021, and in 2022, data collection was not conducted. Therefore, the trendline could not be calculated accurately and has been excluded. Following a decrease from 2016 to 2018, the injury incidence increased between 2018 and 2019 and again between 2019 and 2023 (Figure 3). In 2023, it was the highest recorded to date.

#### U18 Craven Week (CWu18)

The CWu18 tournament was not held in 2020 and 2021 due to COVID-19, and in 2022, there was no data collection; therefore, the trendline could not be calculated accurately and was excluded. Injury incidence gradually increased each year between 2014 and 2017 and then decreased in 2018. In 2019, the injury incidence increased again. In 2023, the injury incidence continued to increase and was the second-highest incidence rate recorded over the 10 years included (Figure 3).

**Figure:**
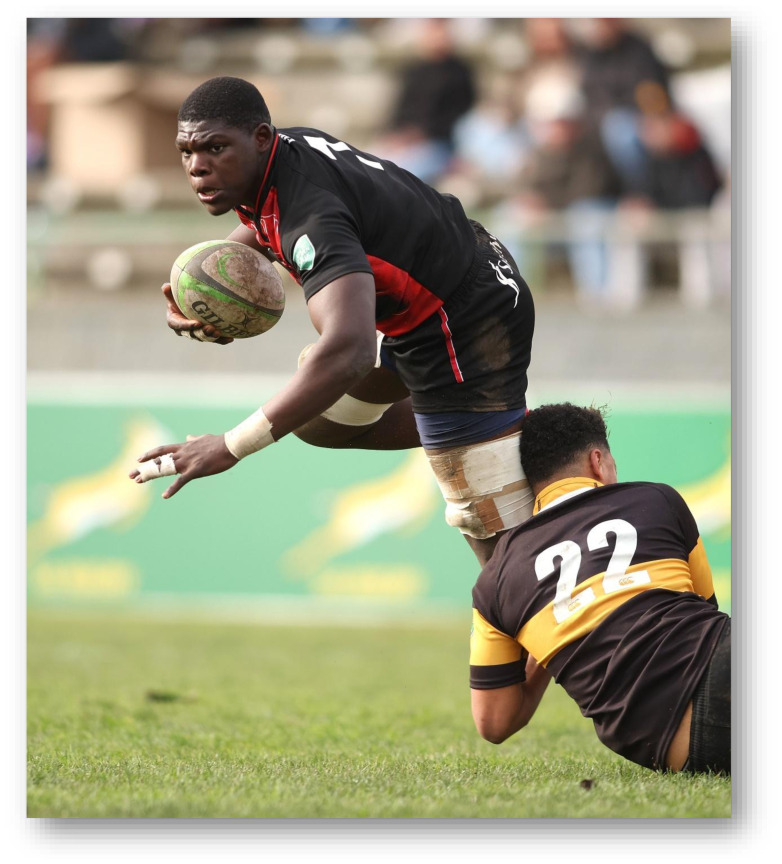


### Injury Event

Considering the combined data from the 2023 tournaments, the Tackler was associated with the most injuries (42%), followed by the Ball Carrier (29%) and Running (10%). Tacklers had 14 (9 to 20) injuries/1000 player hours, while Ball Carriers had 10 (5 to 14) injuries/1000 player hours and Running-related injuries had 3 (1 to 6) injuries/1000 player hours.

Injury incidence to the Tackler was highest in the GKu16 tournament, although the difference was not significant. Ball Carrier and Tackler injury incidence were similar in the CWu18 (Table 3). Running injuries were significantly lower than injuries to the Ball Carriers in the CWu18 tournaments and lower than the Tacklers in the combined data.

**Figure 4:**
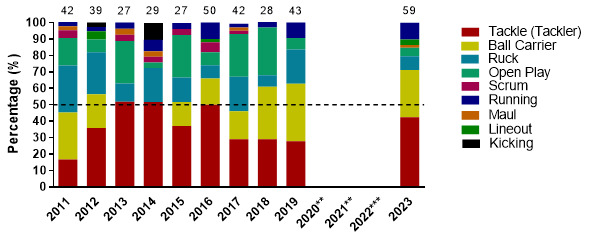
Most common injury-causing events in the GKu16 and CWu18 SARU Boys’ Youth Week tournaments from 2011 – 2023. (The number above each bar represents the total number of injuries for that year). Missing data = 0 cases. **No GKu16 and CWu18 tournaments were held in 2020 and 2021 due to COVID-19 restrictions. *** No data collection was completed in 2022 due to financial constraints.

Figure 4 displays the proportion of injuries resulting from the different injury-causing events between 2011 and 2023. There was an increase in the proportion of injuries sustained by the Tackler from 2019 to 2023. It nonetheless remained lower than the proportion of injuries sustained by the Tackler in 2013, 2014 and 2016.

The Ruck phase of play injuries decreased in percentage from 2019 to 2023. An increase in running-related injuries was noted from 2017 to 2023 (Figure 4).

**Figure 5:**
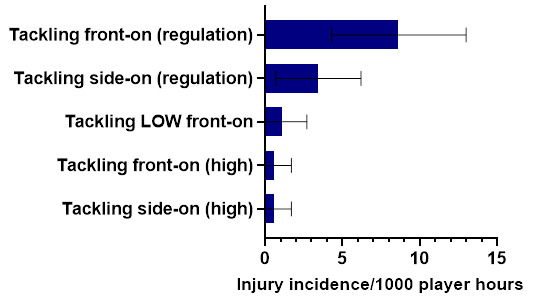
Injury incidence and 95% confidence intervals/1000 player hours of Tackler-related injury mechanisms at the 2023 SARU Boys’ Youth Week Tournaments. Missing data = 0 cases.

In 2023, Tackling front-on (regulation) accounted for the highest proportion of injuries to the Tackler (60%), with 8.6 (4.3 to 13.0) injuries/1000 player hours. Tackling side-on (regulation) accounted for 24% of injuries sustained by the Tackler, with 3.5 (0.7 to 6.2) injuries/1000 player hours (Figure 5).

**Figure 6:**
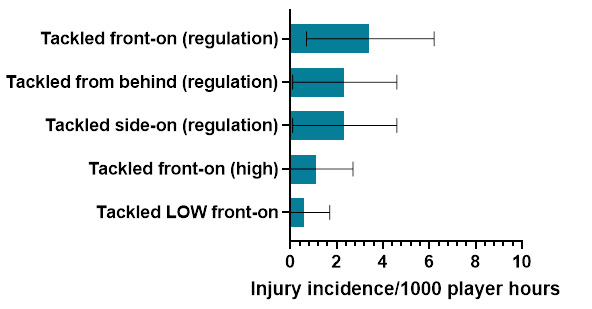
Injury incidence and 95% confidence intervals/1000 player hours of Ball Carrier-related injury mechanisms at the 2023 SARU Boys’ Youth Week Tournaments. Missing data = 0 cases.

Tackled front-on (regulation) accounted for the highest proportion of injuries to the Ball Carrier (35%), with 3.5 (0.7 to 6.2) injuries/1000 player hours (Figure 6).

**Figure 7:**
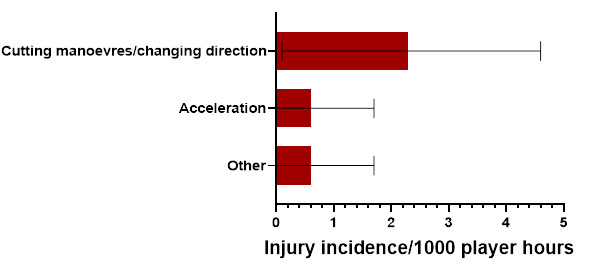
Injury incidence and 95% confidence intervals/1000 player hours of Running-related injury mechanisms at the 2023 SARU Boys’ Youth Week Tournaments. Missing data = 0 cases.

In 2023, Cutting manoeuvres/changing direction accounted for the highest proportion of injuries in the Running-related injuries (67%) with 2.3 (0.1 to 4.6) injuries/1000 player hours. (Figure 7).

**Figure:**
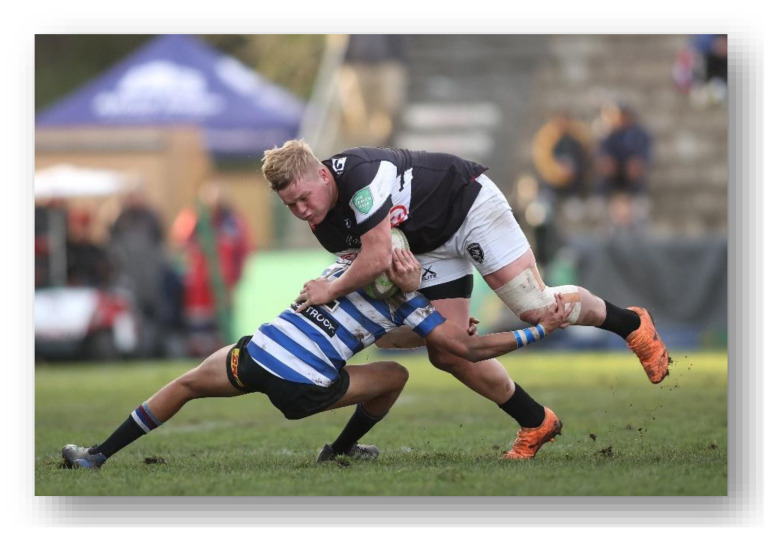


### Injury Type

The most common injury type in the 2023 SARU Boys’ Youth Week tournaments was Central Nervous System (CNS) injuries (Table 4). Although not significant, the combined data across the tournaments showed more CNS injuries than Joint/Ligament and Muscle/Tendon injuries. The GKu16 tournament had the highest incidence of CNS and Joint/Ligament injuries, but again, it was not significantly different from the CWu18.

**Figure 8:**
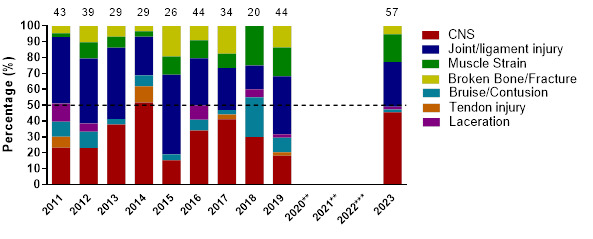
Most common injury types in the GKu16 and CWu18 SARU Boys’ Youth Week tournaments from 2011 – 2023. (The number above each bar represents the total number of injuries for that year). Missing data = 2 cases. **No GKu16 and CWu18 tournaments were held in 2020 and 2021 due to COVID- 19 restrictions. *** No data collection was completed in 2022 due to financial constraints.

Figure 8 illustrates the proportional contribution of the most common injury types per year from 2011 to 2023. CNS injuries contributed more in 2023 than in 2019 but were similar to that recorded in 2017. Joint/Ligament-related injuries were notably less in 2023 than in 2019. Broken Bone/Fractures also decreased noticeably since 2019.

### Body Location

All injuries from the 2023 tournaments were grouped according to the four main body locations (*Head & Neck; Trunk; Upper Body; Lower Body*). In the 2023 tournaments the most common injured body location was the Head & Neck (48%), with 61% of these occurring at the GKu16 tournament. Lower Body injuries were the second most common injured body location. They recorded an injury incidence of 10 (5 to 14) injuries/1000 player hours (Table 5), while the Upper Body injury incidence was 7 (3 to 11) injuries/1000 player hours. Trunk injuries were significantly lower than the other body locations.

Table 6 presents the 2023 SARU Boys’ Youth Week Tissue and Pathology injury data in the format recommended by the most recent IOC consensus statement (2).

### New Vs Recurrent

The injury incidence of *‘New’* injuries in 2023 was 27 (19 to 35) injuries/1000 player hours; a higher injury incidence than in 2019. The subsequent ‘*Recurrent’* injuries were 7 (3 to 11) injuries/1000 player hours, which was similar to 2018.

Most muscle injuries were *‘New’* injuries (88%), and joint/ligament injuries had proportionately more *‘Recurrent’* injuries (31%).

**Figure 9:**
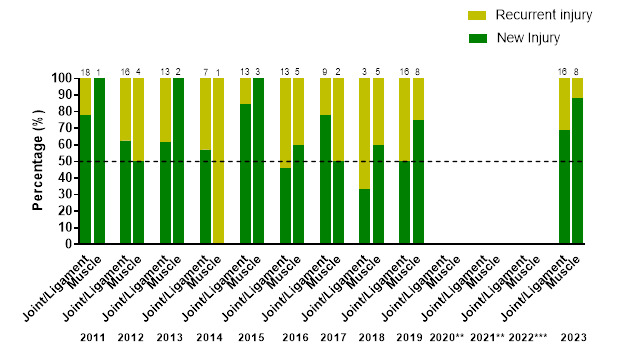
Proportion of New and Recurrent joint/ligament and muscle injuries in the GKu16 and CWu18 SARU Boys’ Youth Week tournaments from 2011 – 2023. (The number above each bar represents the total number of injuries for that year). **No GKu16 and CWu18 tournaments were held in 2020 and 2021 due to COVID-19 restrictions. *** No data collection was completed in 2022 due to financial constraints.

Figure 9 illustrates the proportion of *‘New’* and *‘Recurrent’* joint/ligament, and muscle injuries across the years (2011–2023). There was a slight decrease in the proportion of *‘Recurrent’* joint/ligament injuries from 2019 (50%) to 2023 (31%), and *‘Recurrent’* muscle injuries from 2019 (25%) to 2023 (13%).

There was a corresponding increase in the proportion of *‘New’* joint/ligament injuries from 50% in 2019, to 69%, in 2023. There was also a corresponding increase in the proportion of *‘New’* muscle injuries from 75% in 2019 to 88% in 2023.

### Game Quarter

**Figure 10:**
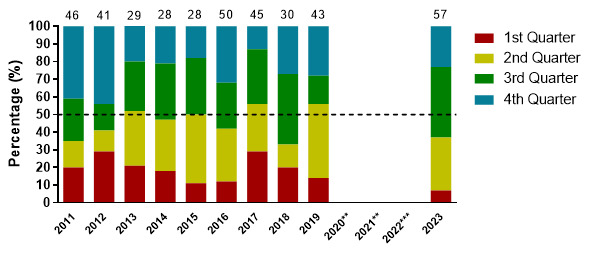
Proportion of injuries occurring in each game quarter in the GKu16 and CWu18 SARU Boys’ Youth Week tournaments from 2011 – 2023. (The number above each bar represents the total number of injuries for that year). Missing data in 2023 = 2 cases. **No GKu16 and CWu18 tournaments were held in 2020 and 2021 due to COVID-19 restrictions. *** No data collection was completed in 2022 due to financial constraints.

Injuries for the 2023 tournaments mainly occurred in the 3^rd^ quarter (40%); 13 (8 to 19) injuries/1000 player hours. Third (3^rd^) quarter injuries increased significantly from 2019 to 2023 (Figure 10).

**Figure:**
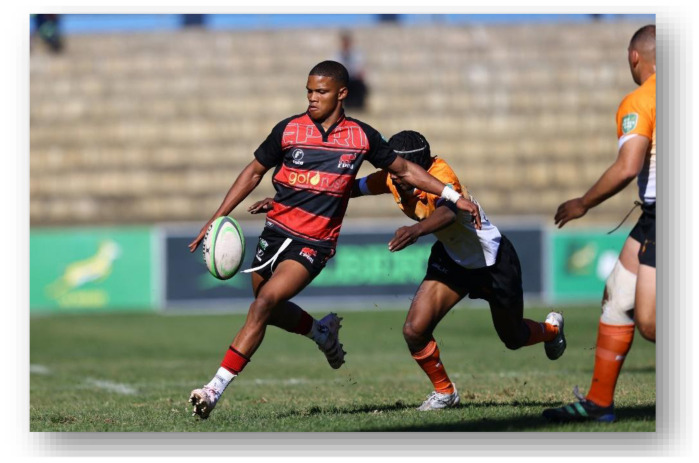


### Match status

**Figure 11:**
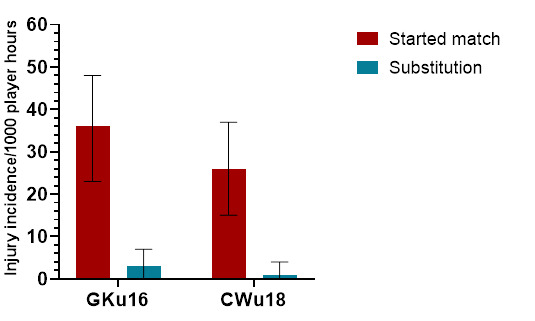
Injury incidence and 95% confidence intervals/1000 exposure hours for players who started the match and those who came on as substitutes, in the 2023 SARU Boys’ Youth Week Tournaments.

In 2023, there were significantly more injuries to players who started the match (93%) compared to players who joined the match as substitutions (7%) (Figure 11). There were no significant differences in injury rates between tournaments for starters or players who came on as substitutes (Table 7).

### Player positions

**Figure 12:**
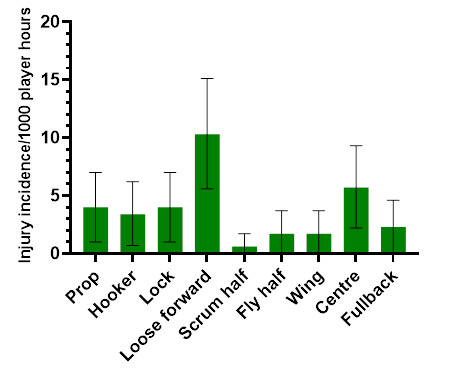
Absolute injury incidence and 95% confidence intervals/1000 player hours per position in the SARU Boys’ Youth Week Tournaments 2023.

In 2023, the loose forward and centre positions had the highest *absolute* injury incidence rates across the SARU Boys’ Youth Week tournaments (Figure 12). Loose forwards and centres had an *absolute* injury incidence of 10.3 (5.6 to 15.1) injuries/1000 player hours and 5.7 (2.2 to 9.3) injuries/1000 player hours, respectively. *Absolute* incidence refers to the incidence of injury in a player’s positional grouping, e.g., wings, without normalising for the number of players on the field playing in that positional grouping, e.g., there are two wings per team on the field.

The number of injuries was also *normalised* to the number of players on the field in a positional grouping. For example, Props = total number of injuries divided by 2, Locks = total number of injuries divided by 2, and Loose forwards = total number of injuries divided by 3.

**Figure 13:**
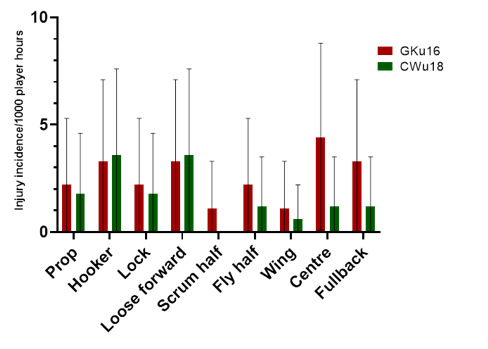
Normalised injury incidence and 95% confidence intervals/1000 player hours per player per position across the two SARU Boys’ Youth Week Tournaments in 2023.

**Figure 14:**
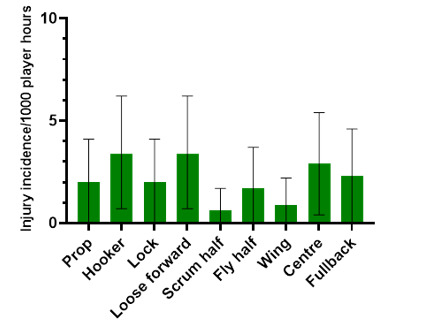
Normalised injury incidence and 95% confidence intervals/1000 player hours per player per position from the combined SARU Boys’ Youth Week Tournaments in 2023.

Figure 13 shows the *normalised* injury incidence per player per position across the two tournaments. In the GKu16, Centres stood out, and in the CWu18, Loose Forwards and Hookers. Figure 14 shows the combined *normalised* positional injury rates from both tournaments. In 2023, when combining the data, the hooker and loose forward positions had the highest *normalised* injury incidence rates. Both hookers and loose forwards, when normalised per player, had an injury incidence of 3.4 (0.7 to 6.2) injuries/1000 player hours (Figure 14).

**Figure:**
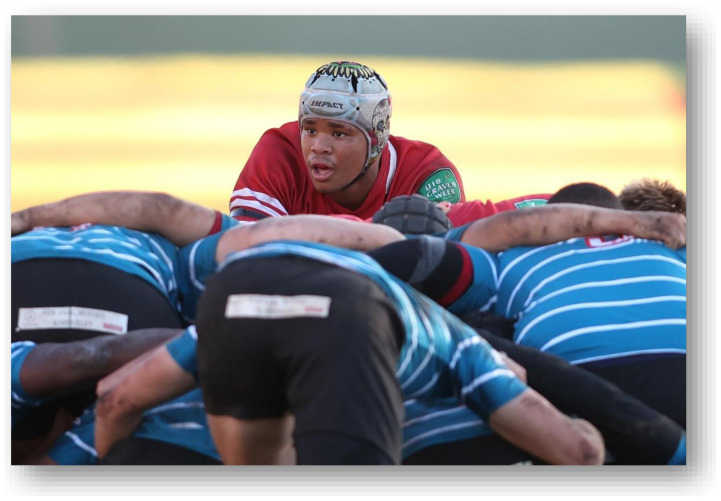


**Figure:**
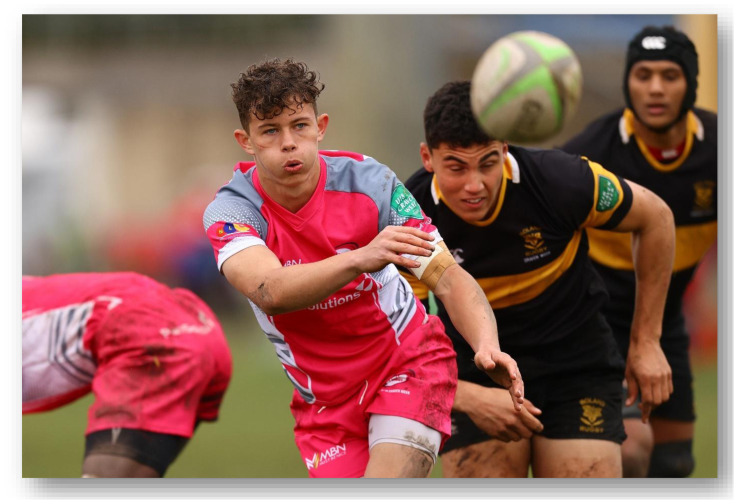


## Concussion

There was a total of 26 concussions recorded over the two tournaments played in 2023, resulting in an incidence rate of 15 (9 to 21) concussions/1000 player hours and approximately one concussion for every two matches played.

The GKu16 was the tournament with the highest concussion incidence rate of 17 (8 to 25) concussions/1000 player hours (Table 8). These data converted to 2 matches per concussion event. The two tournaments concussion rates were not significantly different from each other.

**Figure 15:**
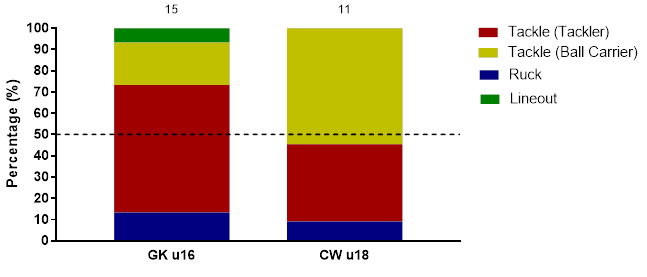
Proportion of concussions caused by the different injury events at the 2023 SARU Boys’ Youth Week Tournaments (n = 26 concussions).

**Figure 16:**
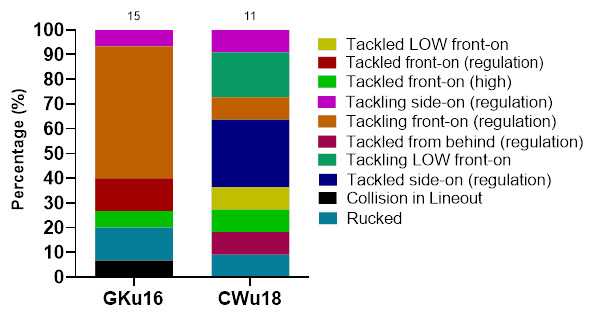
Proportion of concussions caused by the different injury mechanisms at the 2023 SARU Boys’ Youth Week Tournaments (The number above each bar represents the total number of concussions for that Tournament).

In 2023, Tackling (50%, n = 13) and being Tackled (35%, n = 9) were the two events that contributed to the most concussions (Figure 15). Figure 16 displays the proportion of concussions caused by the different injury mechanisms across the tournaments in 2023. Although the numbers are too low to make any firm conclusions, tackling front-on (regulation) had the highest proportion of concussions in GKu16 (53%) and being tackled side-on (regulation) had the highest proportion of concussions for CWu18 (27%).

**Figure 17:**
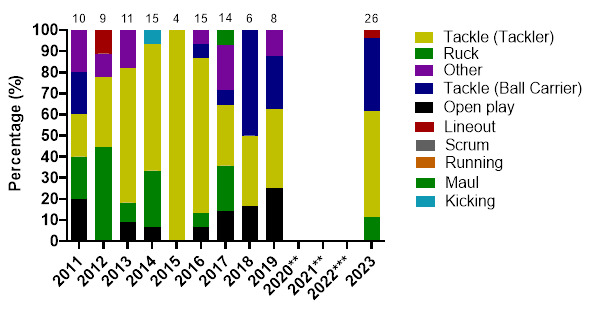
Proportion of concussions caused by the different injury events from 2011 to 2023 *GKu16 and CWu18* SARU Boys’ Youth Week Tournaments. (The number above each bar represents the total number of concussions for that year). **No GKu16 and CWu18 tournaments were held in 2020 and 2021 due to COVID-19 restrictions. *** No data collection was completed in 2022 due to financial constraints.

In Figure 17, the number of concussions increased in 2023. The number of Tackler-related concussions also increased since the period before COVID-19, and the amount of Open Play injury mechanisms causing concussions decreased substantially.

**Figure:**
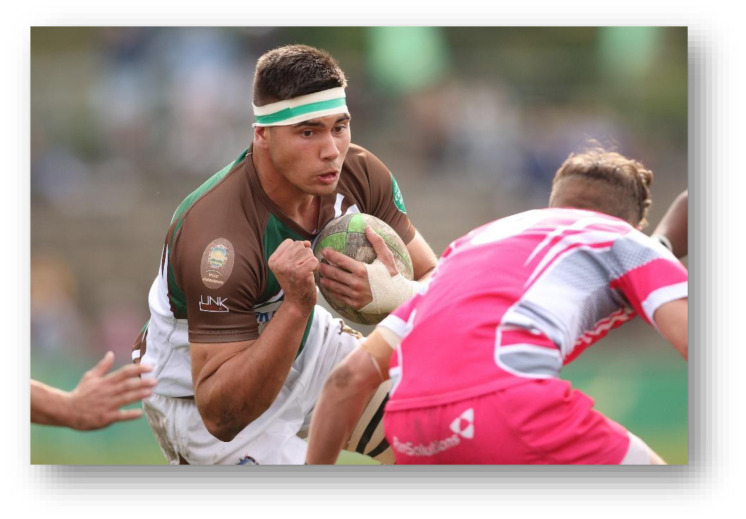


**Figure 18:**
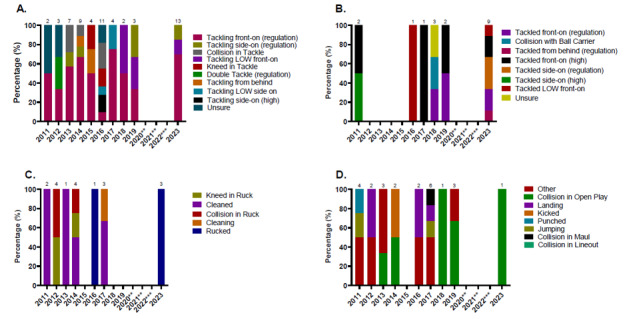
Proportionate breakdown of concussions caused by the various injury mechanisms at the 2011 to 2023 *the GKu16 and CWu18* SARU Boys’ Youth Week Tournaments. (The number above each bar represents the total number of concussions for that year). **A.** Tackler-related concussion mechanisms **B.** Ball Carrier-related concussion mechanisms **C**. Ruck-related concussion mechanisms **D**. Remaining concussion mechanisms. **No GKu16 and CWu18 tournaments were held in 2020 and 2021 due to COVID-19 restrictions. *** No data collection was completed in 2022 due to financial constraints.

Figure 18 shows the percentage of injuries from various mechanisms causing concussions over the ten-year study period. Between 2011 and 2023, 50% of Tackler-related concussions (Figure 18A) were caused by tackling front-on (regulation), 28% of Ball Carrier-related concussions (Figure 18B) were caused by being tackled front-on (high), and 39% of Ruck-related concussions were caused by being cleaned out (Figure 18C).

**Figure 19:**
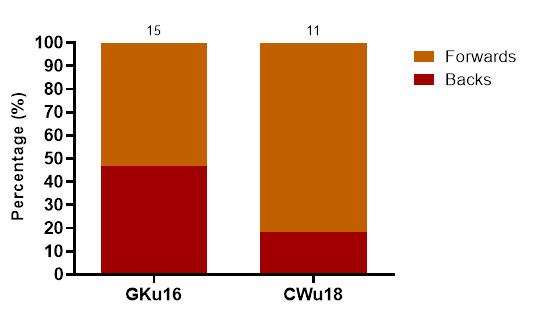
Proportionate breakdown of concussions for forwards and backs at the 2023 SARU Boys’ Youth Week Tournaments (The number above each bar represents the total number of concussions for that tournament).

Most of the concussions in 2023 occurred to forwards (58%). Only 42% of concussions were experienced by backs, most of these occurring in GKu16 (Figure 19).

**Figure 20:**
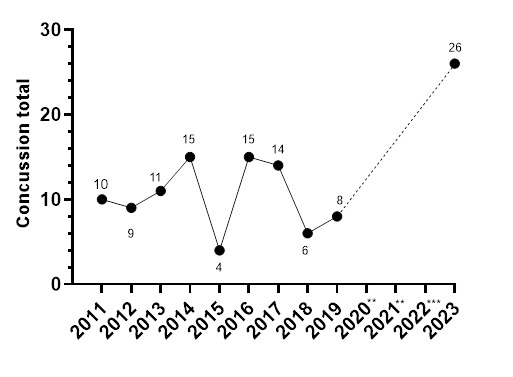
Total number of concussions per year GKu16 and CWu18 SARU Boys’ Youth Week Tournaments from 2011 – 2023. (The number above each data point represents the total number of concussions for that year). **No GKu16 and CWu18 tournaments were held in 2020 and 2021 due to COVID-19 restrictions. *** No data collection was completed in 2022 due to financial constraints.

**Figure 21:**
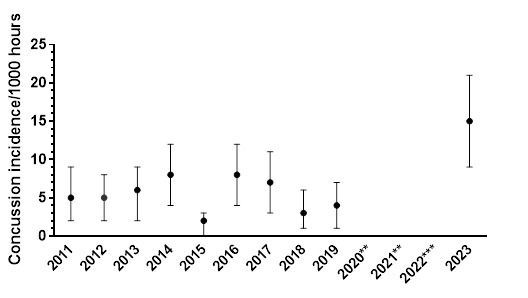
Concussion incidence rates and 95% confidence intervals/1000 player hours per year at the GKu16 and CWu18 SARU Boys’ Youth Week Tournaments from 2011 – 2023. **No GKu16 and CWu18 tournaments were held in 2020 and 2021 due to COVID-19 restrictions. *** No data collection was completed in 2022 due to financial constraints.

From 2011 to 2023, the total number and rate of concussions followed a specific pattern. Both the total number (Figure 20) and rate (Figure 21) for GKu16 and CWu18 initially increased until 2014, sharply decreased in 2015, returned to normal in 2016, and gradually decreased until 2018. However, there was a substantial increase in the concussion rate in 2023, which exceeded expectations. This significant 2023 increase may be attributed to increased awareness of concussions and the disruption in rugby player development and participation in 2020 and 2021 due to COVID-19 (Figures 20 and 21).

**Figure:**
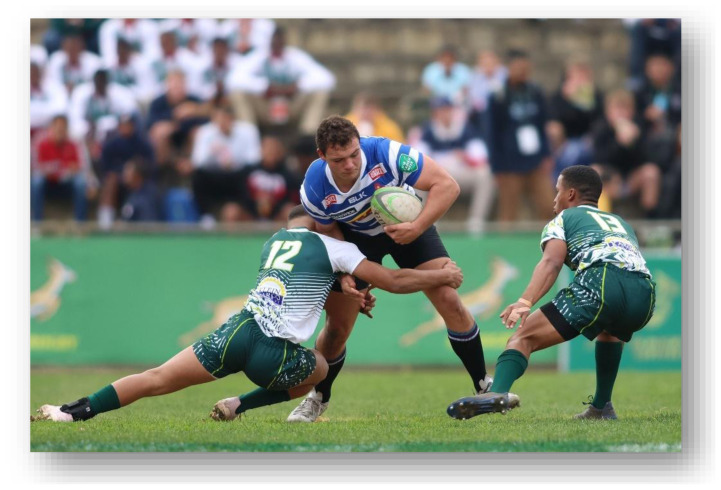


**Figure:**
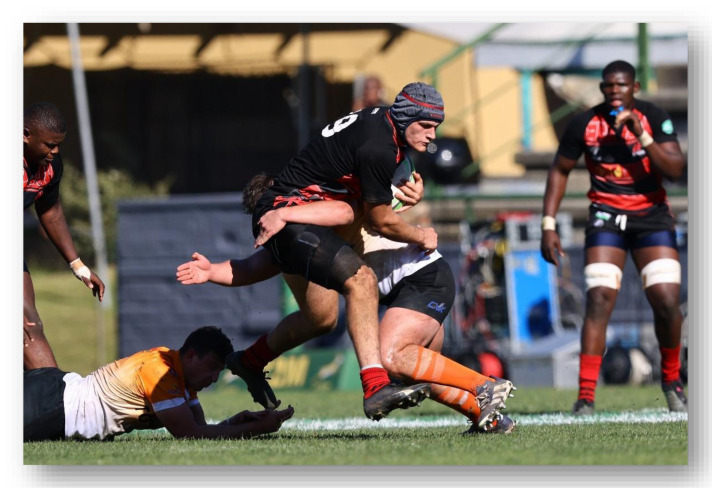


**Figure 22:**
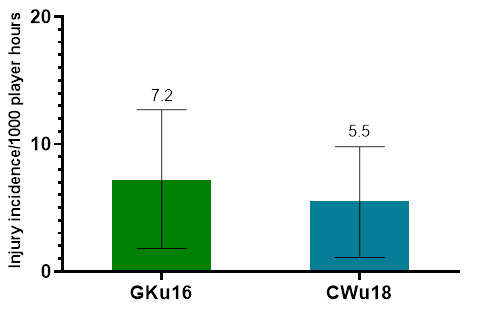
Concussion incidence rates and 95% confidence intervals/1000 player hours per SARU Boys’ Youth Week tournament from 2011 – 2023.

The combined average incidence of concussion injuries (2011 – 2023) decreases as the players’ age and level increase (Figure 22), but these are not significantly different.

**Figure:**
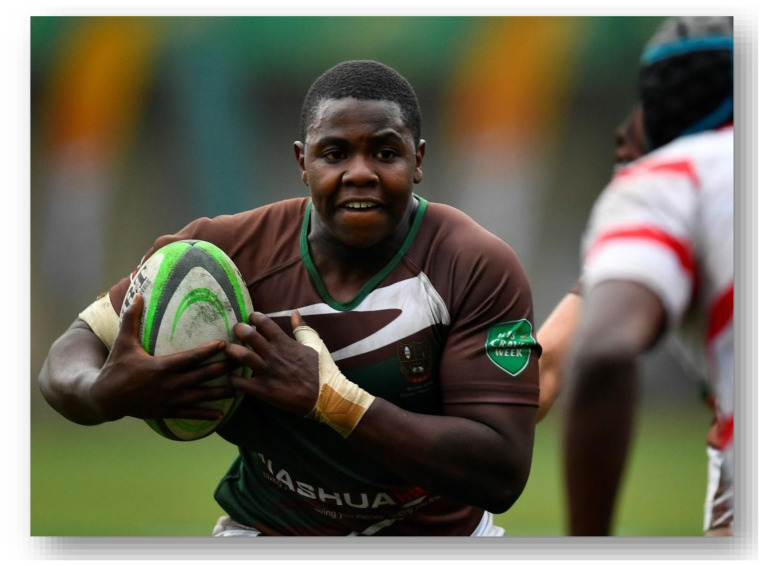


**Figure 23:**
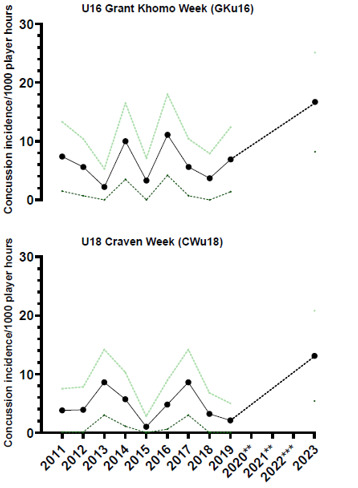
Concussion incidence and 95%CI for each SARU Boys’ Youth Week tournament, from 2011 – 2023. **No GKu16 and CWu18 tournaments were held in 2020 and 2021 due to COVID-19 restrictions. *** No data collection was completed in 2022 due to financial constraints.

The incidence of concussions varied across individual tournaments over time. It initially increased from around 2013 due to improved concussion education, awareness, and stricter protocols, but remained relatively low after 2017 (see Figure 23) until the COVID-19 pandemic. Concussions among GKu16 players increased significantly from 2019, while CWu18 players experienced a decrease from 2017 to 2019 and a similar increase in 2023.

**Figure:**
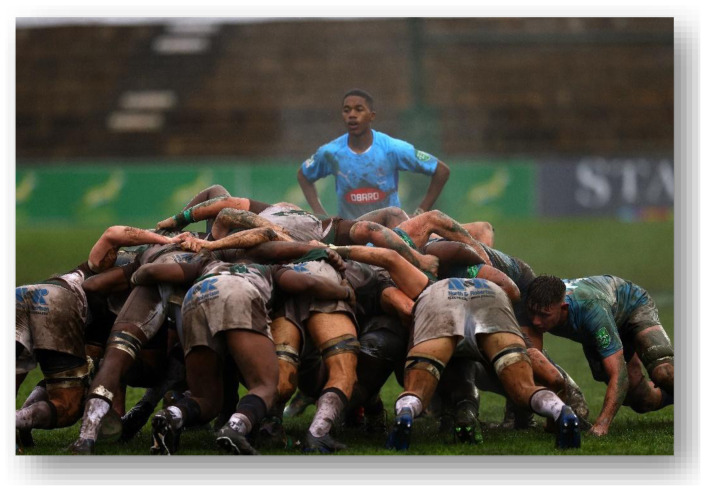


### Take home message

1. The tackle event causes most of the time-loss injuries and concussions.
2. Since most injuries to the tackler and ball carrier occurred with regulation tackles, an emphasis must be placed on developing, teaching and practising the correct, safe, effective tackle and ball carry techniques to minimise these tackle-related injuries.
3. Following the recent SARU law change of lowering the legal tackle height, contact technique and correct body position has gained importance for continued injury prevention.
4. Players in positions that may carry a higher risk of injury and require more contact than other positions need to be appropriately selected, adequately conditioned, and physically prepared to play in these roles; for example, Hookers and Loose forward.
5. Incorporating focused strength training, physical conditioning, and agility drills can help prevent running-related injuries, particularly those associated with cutting manoeuvres and changes of direction
6. Coaches and their medical support staff must ensure that players are properly rehabilitated after an injury before they return to play. There is a concern about potentially returning players to the field too early following an injury, especially at this level of play.

**Figure:**
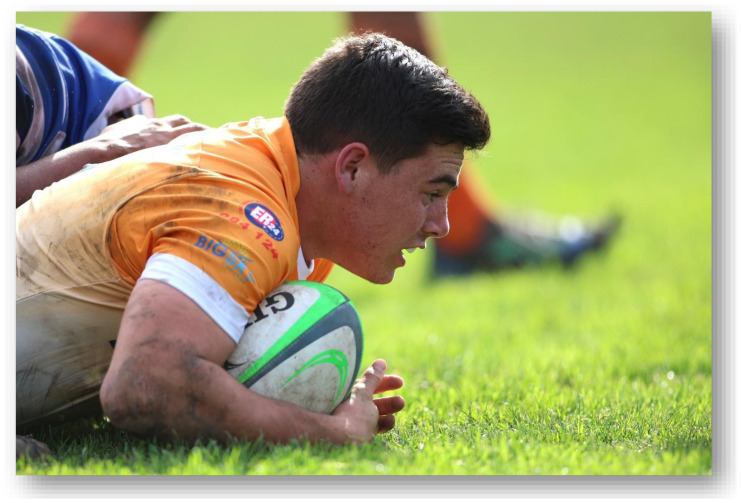


**Figure:**
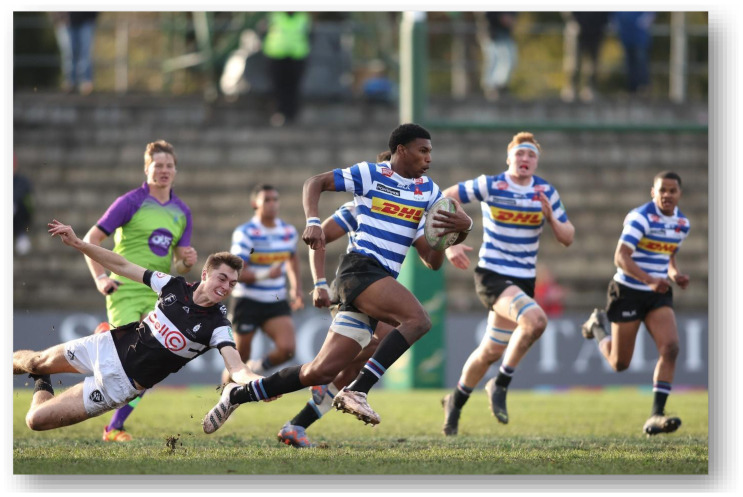


## Figures and Tables

**Table 1:** Number and injury incidence (95% CI)/1000 player hours of Medical Attention and Time-Loss injuries in the 2023 SARU Boys’ Youth Week tournaments.

	Medical Attention Injuries		Time-Loss Injuries	

	Number	Incidence	Number	Incidence
**GKu16**	42	47 (33 – 61)	36	40 (27 – 53)
**CWu18**	46	55 (39 – 71)	23	27 (16 – 39)

***Combined Total***	***88***	***51 (40 – 61)***	***59***	***34 (25 – 43)***

**Table 2:** Number of Medical Attention and Time-Loss injuries. Data expressed per match and per hour played in the 2023 SARU Boys’ Youth Week tournaments.

Tournament	Number of matches	Match duration (mins)	Medical Attention injuries/match	Time-Loss injuries/match	Medical Attention injuries/hour	Time-Loss injuries/hour
**GKu16**	30	60	1.4	1.2	1.4	1.2
**CWu18**	24	70	1.9	1.0	1.6	0.8

***Combined Tournament Average***	***27***	***65***	***1.7***	***1.1***	***1.5***	***1.0***

**Table 3:** Injury incidence (95% CI)/1000 player hours of Time-Loss injuries to the Tackler roles, Ball Carrier roles, and Running injuries for the 2023 SARU Boys’ Youth Week tournaments.

Tournament	Tackler	Ball Carrier	Running
**GKu16**	19 (10 – 28)	8 (2 – 14)	6 (1 – 10)
**CWu18**	10 (3 – 16)	12 (5 – 19)	1 (0 – 4)*

***Combined total***	***14 (9 – 20)***	***10 (5 – 14)***	***3 (1 – 6)*** **

* Significantly lower than CWu18 Ball Carriers

** Significantly lower than Combined total Tacklers

**Table 4:** Injury incidence (95% CI)/1000 player hours of Time-Loss injuries at the 2023 SARU Boys’ Youth Week tournaments grouped as Central Nervous System (CNS), Joint/Ligament, and Muscle/Tendon injuries.

Tournament	CNS	Joint/Ligament	Muscle/Tendon
**GKu16**	17 (8 – 25)	12 (5 – 19)	4 (0 – 9)
**CWu18**	13 (5 – 21)	6 (1 – 11)	7 (1 – 13)

***Combined Total***	***15 (9 – 21)***	***9 (5 – 14)***	***6 (2 – 9)***

**Table 5:** Proportion (%) and incidence (95% CI)/1000 player hours of Time-Loss injuries, grouped by body location, in the 2023 SARU Boys’ Youth Week tournaments.

	Proportion of injuries (%)	Incidence (95% CI)/1000 player hours
Head & Neck	48	16 (10–22)
Lower Body	29	10 (5–14)
Upper Body	20	7 (3–11)
Trunk	3	1 (0–3)*

* Significantly lower than the other grouped Body Locations

**Table 6:** Injuries grouped according to the IOC recommended categories of Tissue and Pathology types for the 2023 SARU Boys’ Youth Week tournaments.

Tissue	Incidence	Mean time loss

*Pathology*	*Injuries per 1000 hours (95% CI)*	*Days (95% CI)*
**Muscle/Tendon**	**6 (2 to 9)**	**11 (5 to 16)**
*Muscle injury*	6 (2 to 9)	**11 (5 to 16)**
**Nervous**	**15 (9 to 21)**	**14**
*Concussion*	15 (9 to 21)	14
**Bone**	**2 (0 to 4)**	**25 (14 to 35)**
*Fracture*	1 (0 to 3)	30
*Bone contusion*	1 (0 to 2)	14
**Ligament/Joint Capsule**	**9 (5 to 14)**	16 (11 to 20)
**Superficial tissue/skin**	**1 (0 to 2)**	11 (4 to 17)
*Contusion (superficial)*	1 (0 to 2)	7
*Laceration*	1 (0 to 2)	14
Other*	**1 (0 to 2)**	**7**

*TOTAL*	**34 (25 to 43)**	**14 (13 to 16)**

Estimated severity for Time-Loss was used from data provided by the Tournament Doctors at the venue when real-time severity was not able to be determined.

* All Other injuries were soft tissue injuries. Due to the nature of the data collection process, we were unable to delve deeper into determining a specific diagnosis.

**Table 7:** Number of injuries and injury rates (95% CI)/1000 exposure hours for players who started the match and those who came on as substitutes in the 2023 SARU Boys’ Youth Week tournaments. Missing data = 1 case.

	Started match		Substitution	

	Number	Incidence	Number	Incidence
**GKu16**	32	36 (23–48)	3	3 (0–7)*
**CWu18**	22	26 (15–37)	1	1 (0–4)*

***Combined Total***	***54***	***31 (23–39)***	***4***	***2 (0–5)*** *

* Significantly lower than players who started matches

**Table 8:** Number and incidence of concussions (95% CI)/1000 player hours at the 2023 SARU Boys’ Youth Week tournaments.

Tournament	Number	Incidence	Number of matches per concussion event
**GKu16**	15	17 (8 – 25)	2
**CWu18**	11	13 (5 – 21)	2

***Combined Total***	***26***	***15 (9 – 21)***	***2***

## References

[1] Fuller CW, Molloy MG, Bagate C (2007). Consensus statement on injury definitions and data collection procedures for studies of injuries in rugby union. Br J Sports Med.

[2] Bahr R, Clarsen B, Derman W (2020). International Olympic Committee consensus statement: methods for recording and reporting of epidemiological data on injury and illness in sport 2020 (including STROBE Extension for Sport Injury and Illness Surveillance (STROBE-SIIS)). Br J Sports Med.

[3] Fuller CW (2017). A Kinetic model describing injury-burden in team sports. Sport Med.

[4] Schenker N, Gentleman JF (2001). On judging the significance of differences by examining the overlap between confidence intervals. Am Stat.

